# Circular RNA expression is suppressed by androgen receptor (AR)-regulated adenosine deaminase that acts on RNA (ADAR1) in human hepatocellular carcinoma

**DOI:** 10.1038/cddis.2017.556

**Published:** 2017-11-16

**Authors:** Liang Shi, Peijian Yan, Yuelong Liang, Yin Sun, Jiliang Shen, Senjun Zhou, Hui Lin, Xiao Liang, Xiujun Cai

**Affiliations:** 1 Key Laboratory of Endoscopic Technique Research of Zhejiang Province, Department of General Surgery, Sir Run Run Shaw Hospital, Zhejiang University, Hangzhou 310016, China; 2 Department of Radiation Oncology, University of Rochester Medical Center, Rochester, NY 14642, USA

## Abstract

Hepatocellular carcinoma (HCC) is a heterogeneous malignancy as a result of complex genetic and epigenetic alterations. HCC is characterized by a clear gender disparity for which there is lack of a clear mechanistic understanding. Androgen receptor (AR) is thought to be critical for such bias. Meanwhile, the potential function of circular RNA (circRNA), regulated by RNA editing enzyme, remained largely unknown in malignancy till now. By utilizing circRNA microarray survey coupled with *in vitro* analysis, we analyzed the influence of AR on circRNA expression in HCC. Our results indicated that AR could suppress circRNA expression by upregulating ADAR1 p110. Such effect is because AR served as a transcriptional activator of ADAR1 promoter. More significantly, data collected from our center strongly suggest that ADAR1 expression can effectively predict HCC patients’ prognosis and an abnormal overexpression of ADAR1 is positively correlated with AR in HCC. In addition, we found CircARSP91 (hsa_circ_0085154), one of the circRNAs downregulated by AR in an ADAR1-dependent manner, could inhibit HCC tumor growth both *in vitro* and *in vivo.* These findings highlight the fact that AR as a contributing factor for gender disparity in HCC can cause complex consequences though regulation of circRNA expression. Better understanding of the roles of circRNA during HCC initiation and progression will provide a novel angle to develop potential HCC therapies.

Hepatocellular carcinoma (HCC), accounting for almost 80% of all the primary liver cancers, is the second leading cause for cancer-related death worldwide.^1^ In 2012 alone, an estimated 745 000 patients died of HCC.^2^ In our center, only limited number of HCC patients qualified for primary resection or liver transplantation as a curative procedure for HCC treatment, and similar dilemma is encountered all over the world.^3, 4^ Salvage chemotherapy has limited options with the recent addition of Sorafenib as the only one proved by the Food and Drug Administration in United States.^5, 6^

Many attempts, including hormone therapy,^7, 8^ were made to develop novel therapies to more effectively control HCC with unsatisfactory outcomes. A better understanding of the HCC initiation and progression will likely provide novel and efficacious treatment for HCC.^9^ The role of androgen receptor (AR) in gender disparity of HCC during initiation and progression has been well documented, yet application of that knowledge has not been successful in providing therapies for HCC.^10, 11^

Recently, circular RNAs (circRNAs) emerged as potential molecules mediating tumor biology and function through multiple mechanisms.^12, 13, 14, 15, 16^ However, the functions of circRNAs in HCC remained poorly understood.^17^ Meanwhile, adenosine deaminases that act on RNA (ADAR1) has been well studied in HCC as a tumor promoter by interrupting RNA editing equilibrium.^18^ Intriguingly, ADAR1 was also reported to be a critical regulator of circRNA formation.^19^

In this study, we first revealed that AR differentially regulated circRNA expression in HCC through ADAR1 that could directly suppress RNA circularization.^19^ Moreover, we found AR influenced expression of circRNAs significantly among those originated from polyadenylate-binding protein 1 gene (PABPC1) and indeed one of them, hsa_circ_0085154, could regulate HCC proliferation *in vivo*.

## Results

### Androgen receptor suppressed circular RNA expression in HCC

**Figure 1:**
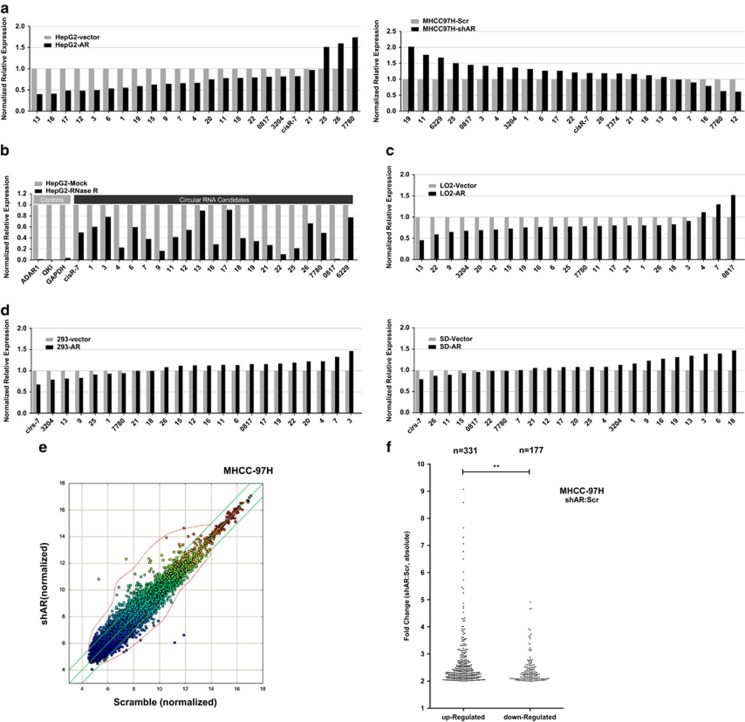
AR suppresses circRNA expression in HCC cell lines. (**a**) Overexpressed (left panel) and knocked-down AR (right panel) changed expression of a cohort of selected circRNAs in HCC cells, detected by RT-qPCR. The lists of circRNAs may have some difference because some circRNAs could not be detected in certain cell lines. (**b**) Extracted RNA was treated by RNase R before reverse transcription. Then, the RT-qPCR was performed and verified that these selected circRNAs were indeed circular. (**c**) RT-qPCR revealed that AR suppressed expression of these circRNAs in normal liver LO2 cells. (**d**) RT-qPCR revealed that AR failed to suppress expression of these circRNAs in non-HCC cells. Left, human embryonic kidney 293T cells; right, gall bladder cancer SD cells. (**e**) Scatter plot for a visualization of the CircRNA expression variation. The values of *x* and *y* axes in the scatter plot are the normalized signal values of the samples (log2 scaled) or the averaged normalized signal values of groups of samples (log2 scaled). The green lines are fold change lines. The CircRNAs above the top green line and below the bottom green line indicated more than twofold change of circRNAs between the two compared samples. Area between red line and upper or lower green lines indicated the major distribution range of differentially expressed circRNAs. (**f**) Among knocking down AR modulated circRNAs, boosted ones are outnumbered than suppressed ones (331 to 177). **P<0.01

In an attempt to examine whether AR as a transcription factor^20^ can regulate circRNA expression in HCC, we selected all the verified circRNAs from two previous publications^21, 22^ and the host gene of these circRNAs would not affected by AR. Then, we examined their expression through quantitative real-time PCR (RT-qPCR) in response to AR expression in different HCC cells (Figure 1a and Supplementary Figure S1d). The results revealed that AR uniformly suppressed expression of this panel of circRNAs whether in the form of overexpressed (oe) or knocked down (KD or sh) AR. To verify the reliability of this circRNA panel, we used RNase R that has a higher activity toward linear RNA than circRNA to treat the samples. The results confirmed the primers we used could specifically detect these circRNAs (Figure 1b and Supplementary Figure S1e). Similar results were also obtained when we replaced tumor cells with LO2, a hepatocyte cell line^23^ (Figure 1c). However, we did not observe this phenomenon in 293T cells and gall bladder cancer cells (Figure 1d). These results suggested that AR could specifically regulate in liver cells expression of a large number of circRNAs, but it is not clear whether it is via a transcriptional or post-transcriptional mechanism.

### Human circular RNA microarray screening verified the role of AR in circular RNA expression

In order to extend the number of potential target circRNAs regulated by AR, we analyzed the circRNA expression with a circular RNA array. We used an AR-high expressing HCC cell line MHCC-97H to perform AR KD and then extracted total RNA for the array analysis with a total of 13 199 circRNA candidates on the chip (Supplementary Figure S1f). The results identified a total of 508 dysregulated circRNAs in AR-KD groups compared with the control groups, with a fold change ≥2.0, *P*<0.05 and FDR <0.05. Among these differentially expressed circRNAs, 331 were upregulated whereas only 177 were downregulated (Figure 1e, fold change ≥2.0, *P*<0.05 and FDR <0.05). Also, the fold change is larger for the upregulated circRNAs than for the downregulated ones (Figure 1f). We also tested the correlation between measurements by microarray and RT-qPCR. The result revealed that two different approaches reached a similar conclusion (Figure 1a right panel and Supplementary Figure S1g). Intriguingly, we observed a much smaller change in circRNA expression in normal liver cells when AR was manipulated (Supplementary Figures S1h and i). This result confirmed our hypothesis that AR could globally suppress circRNA expression in HCC.

### AR repressed circRNA expression via upregulating ADAR1

**Figure 2:**
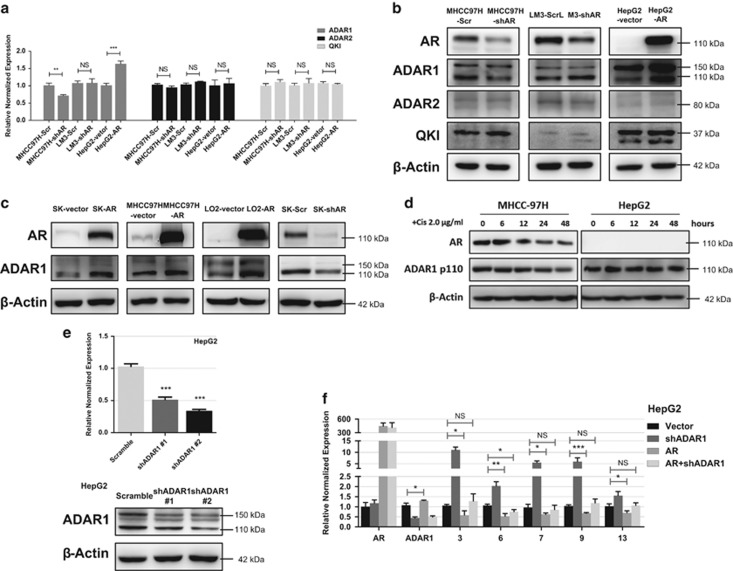
AR modulates circRNA expression by upregulating ADAR1 p110. (**a**–**c**) Regulation of protein implicated in circRNA expression by AR. ADAR1 p110 appears to be regulated by AR at both mRNA (RT-qPCR) and protein levels (western blots). (**d**) After cisplatin treatment, western blot results indicated that ADAR1 p110 decreased in AR-positive HCC cells compared with no change in AR-negative cells and the dosage was determined based on a previous report.^25^ (**e**) Knockdown efficiency of shADAR1 plasmid. Upper panel indicates RT-qPCR results and lower western blots. (**f**) We performed rescue assays by knocking down ADAR1 in AR-overexpressed cells and then detecting selected circRNAs with RT-qPCR. Data shown are mean±S.D. *P<0.05, **P<0.01 and *** *P*<0.001

AR can suppress the expression of a large number of circRNAs, suggesting that AR can suppress the expression of the host genes for those circRNAs or AR can regulate the expression of circRNAs through a post-transcriptional mechanism. AR is generally considered as a transcriptional activator, although in prostate cancer AR can also repress gene expression, and thus we believe it is more likely that AR functions through the latter mechanism to influence circRNA expression in HCC. Indeed, it has been reported that regulatory proteins, including ADAR1 and QKI, could participate in the formation of circRNAs and thus global circRNA expression.^19, 24^ Therefore, it is possible that AR may suppress circRNA expression via regulating these proteins directly or indirectly. To test that, we measured mRNA and protein expression of these molecules after manipulating AR in three HCC cell lines (Figures 2a and b). We found in two cell lines, MHCC-97 and HepG2, that AR promoted ADAR1 p110 expression at both mRNA and protein levels whereas LM3 cells did not. On the other hand, ADAR1 p150, ADAR2 and Quaking (QKI) remained unchanged in all three cell lines. We also used western blot analysis with more cell lines to determine ADAR1 p110 expression and examined its relationship to AR (Figure 2c), and the results were consistent with the earlier finding that AR increased ADAR1 expression. In addition, Cisplatin treatment of HCC cells led to a decrease of ADAR1 p110 consistent with our previous report that Cisplatin could suppress AR expression,^25^ whereas Cisplatin failed to do so in AR-negative cells (Figure 2d). We also tested the effect of androgen in our system by artificially removing androgen from culture media then added back. The results indicated androgen could increase ADAR1 p110 expression in AR-positive cells (SK-Hep1 and MHCC-97H) whereas ADAR1 p110 remained intact in AR-negative cells (HepG2), suggesting androgen could function through AR to influence ADAR1 p110 (Supplementary Figure S2a).

ADAR1 was found to be a strong suppressor during circRNA production and its A-to-I editing process occurred more frequently near the location of reverse complementary matches (RCMs), a structure believed to be critical for circRNA formation.^19^ In order to test whether ADAR1 is indispensable for AR-induced circRNA repression, we performed ADAR1 KD in AR-overexpressed cells and detected circRNA changes with RT-qPCR. The ADAR1 KD efficacy is shown in Figure 2e and we used sh-RNA #2 for the rest of experiments. The results suggested that KD ADAR1 in AR-positive cells could partially rescue expression of repressed circRNAs (Figure 2f and Supplementary Figure S2b), consistent with the notion that AR can regulate circRNA expression through regulating ADAR1 expression.

### ADAR1 is abnormally elevated and associated with AR expression in HCC

**Figure 3:**
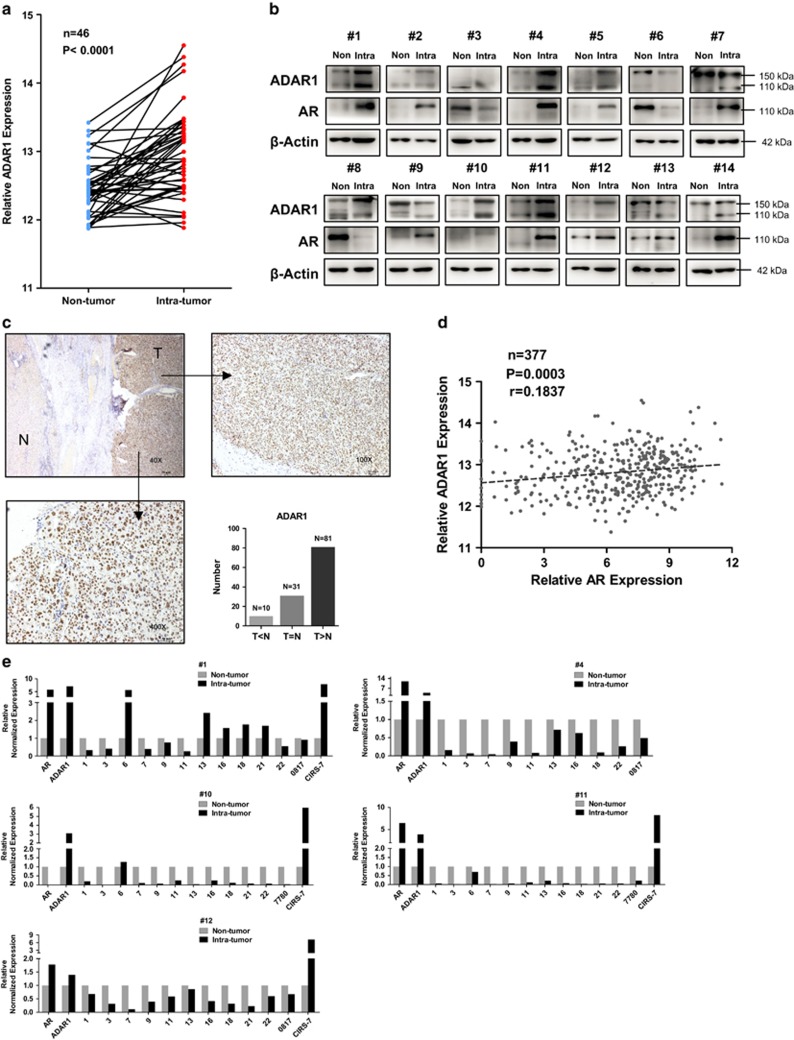
Abnormal ADAR1 expression in HCC and its correlation with AR. (**a**) Data extracted from TCGA showed ADAR1 is predominantly overexpressed in HCC tumor tissues compared with paired normal tissues. (**b**) Western blots of HCC samples collected from our center showed ADAR1 and AR were overexpressed in tumors. (**c**) IHC results indicated that ADAR1 was significantly elevated in HCC tumor tissue compared with adjacent normal tissue. Upper and left lower pictures are representative images of IHC staining for ADAR1 (T, tumor tissue; N, normal liver tissue), and right lower is quantitative result for case numbers (T, ADAR1 level in tumor tissue; N, ADAR1 level in normal live tissue). (**d**) Pearson’s correlation analysis of TCGA HCC data revealed a positive correlation between AR and ADAR1. (**e**) RT-qPCR analysis of circRNAs expression in patient samples indicated that most circRNAs were downregulated in tumor tissues. The lists of circRNAs may have some difference because some circRNAs could not be detected in certain cell lines. Non, nontumor tissue; Intra, intratumor tissue; *n*, case number; *r*, Pearson’s correlation coefficient; *P*, *P-*value

Unbalanced A-to-I editing by ADAR1 was identified to be a tumor promoter in various tissues.^26^ In particular, the abnormal expression of ADAR1 promoted HCC development.^18, 27^ By analyzing the TCGA database (http://cancergenome.nih.gov/), we confirmed that ADAR1 was elevated in HCC tumor tissues compared with adjacent normal tissues (Figure 3a). Analysis of Oncomine database with results obtained from different groups also supported this conclusion (Supplementary Figures S2c–g). This general conclusion was further supported by our own analysis with patient samples from our center of western blot and immunohistochemistry (IHC) of ADAR1 expression (Figures 3b and c). In order to detect potential ADAR1 expression by AR in patient samples, we did the Pearson test on the 377 cases from the TCGA data set and found a weak but solid positive correlation between AR and ADAR1 (Figure 3d), and this relationship also holds in our own HCC samples (Figure 3b). These results support the conclusion that ADAR1 expression promotes HCC development, likely as a result of overactivation of AR.

### CircRNAs dramatically decreased in tumor compared with normal liver tissue

In order to examine the potential biochemical consequences of ADAR1 overexpression, we examined the expression of limited number of circRNAs in HCC patient samples, and found that expression of circRNAs in HCC tumor tissue is generally lower than that in adjacent normal tissue except for CIRS-7 (Figure 3e). These results thus supported the conclusion that higher ADAR1 expression likely will result in a lower expression of many circRNAs in HCC.

### Higher ADAR1 expression level is correlated with a worse prognosis in HCC

**Figure 4:**
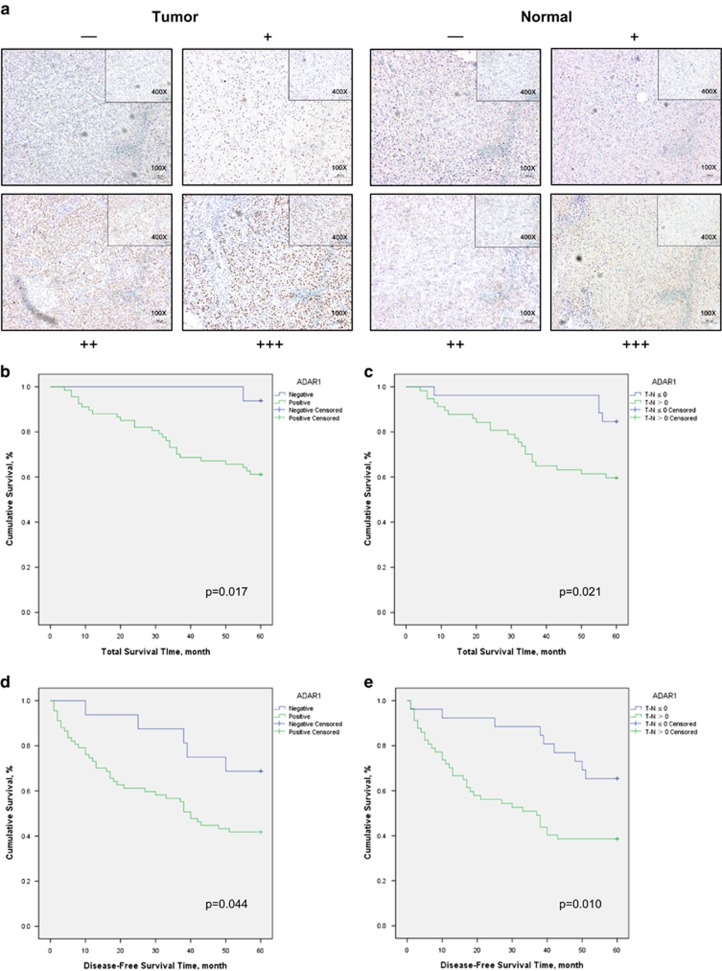
Prognostic value of ADAR1 expression in HCC after tumor resection. (**a**) Representative images for scoring the ADAR1 IHC staining in HCC tumor tissues (left panel) and normal liver tissues (right panel). (**b** and **c**) Kaplan–Meier plots for total survival time of patients demonstrating ADAR1-negative (blue line in **b**; *n*=25), ADAR1-positive (green line in **b**; *n*=58), ADAR1 normal expression or downregulation (blue line in **c**; *n*=32) and ADAR1 overexpression (OE) (green line in **c**; *n*=51) in tumor tissues (log-rank test). (**d** and **e**) Kaplan–Meier plots for disease-free survival time of patients demonstrating ADAR1-negative (blue line in **d**; *n*=25), ADAR1-positive (green line in **d**; *n*=58), ADAR1 normal expression or downregulation (blue line in **e**; 32) and ADAR1 overexpression (OE) (green line in **e**; *n*=51) in tumor tissue (log-rank test). T, tumor; N, nontumor

An oncogenic role of ADAR1 in HCC progression was reported with measurement of ADAR1 mRNA through semiquantitative real-time PCR.^18^ As the transcript level may not always represent the protein level in the tissue, we decided to repeat survival analysis with patients acquired from our center using IHC scoring for ADAR1 expression. We collected a panel of 83 patients with surgically resected primary HCC and their matched normal liver tissues from Sir Run Run Shaw Hospital, and performed IHC staining for ADAR1 intratumor and nontumor (NT) expression and scored its immunoreactivities as negative (−), weak positive (+), moderate positive (++) and strong positive (+++) (Figure 4a). Kaplan–Meier plots for total survival time revealed a worse outcome in ADAR1 intra-tumor-positive (+, ++ and +++) patients compared with the negative group (−) (Figure 4b). Interestingly, when the follow-up period extends from 60 to 120 months, the difference loses significance (Supplementary Figure S3a). However, if the difference of ADAR1 expression between intratumor and nontumor is included, the abnormal increase of ADAR1 in tumor tissues promoted cancer progression significantly up to 120 months (Figure 4c and Supplementary Figure S3b). Similarly, this relationship extended to the disease-free survival time, and thus these results strongly suggested that ADAR1 could facilitate HCC recurrence and progression (Figures 4d and e and Supplementary Figures S3c and d). Unfortunately, we could not reach a statistical significance correlating ADAR1 expression grade with both total survival time and disease-free survival time (Supplementary Figures S3e and F).

### AR transcriptionally up-regulates ADAR1 p110 via binding to its promoter

**Figure 5:**
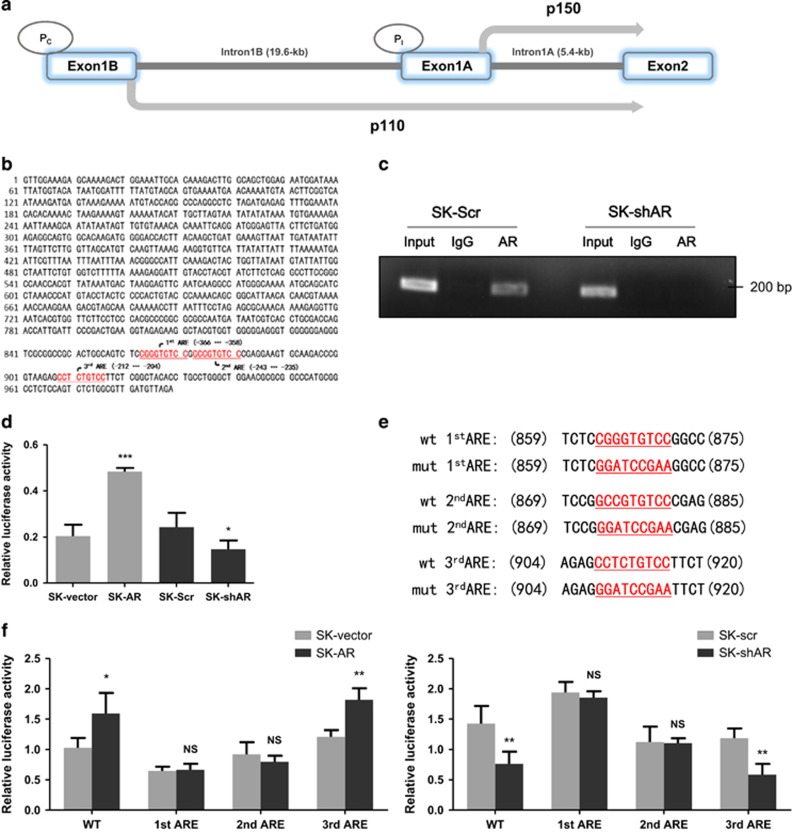
AR activates ADAR1 p110 transcription by binding to its promoter region. (**a**) Schematic diagram for promoters’ structure of ADAR1 p150 and p110. (**b**) Predicted localization of AREs in ADAR1 p110 promoter region (red). (**c**) Chromatin immunoprecipitation was performed in SK-Hep1 cells after manipulating AR with shRNA. The detecting primer was designed based on the prediction result of potential AREs. (**d**) Wild-type ADAR1 p110 promoter construct was transfected into SK-Hep1 cells with internal control pRL-TK. Then, we performed luciferase reporter assays with manipulated AR to detect if AR could affect activation of ADAR1 p110 promoter. (**e**) A scheme of mutated AREs by quickchange in luciferase reporter plasmid. (**f**) Luciferase reporter assays were performed after transfecting three mutated AREs respectively in AR-overexpressed SK-Hep1 cells (left panel) and AR knocked-down SK-Hep1 cells (right panel). Data shown are mean±S.D. *P<0.05, ** *P*<0.01 and *** *P*<0.001. P_I,_ IFN-inducible ADAR1 promoter; P_C,_ constitutively active promoter

ADAR1 has at least two different promoters from which p150 and p110 originates respectively.^28, 29^ One of them was identified as an IFN-inducible ADAR1 promoter (P_I_) and transcription of ADAR1 full-length p150 starts from exon1A. The second promoter (P_C_) was thought to be constitutively active and responsible for coding of ADAR1 p110 from exon1B (Figure 5a). We analyzed the promoter region (http://www.genecards.org/cgi-bin/carddisp.pl?gene=ADAR) of ADAR1 p110 isoform with ALGGEN-PROMO software (http://alggen.lsi.upc.es/cgi-bin/promo_v3/promo/promoinit.cgi?dirDB=TF_8.3) and identified several potential AR-responsive elements (AREs) (Figure 5b). Next we performed chromatin immunoprecipitation (ChIP) assays and verified AR could bind to this region (Figure 5c). In a reporter assay, AR activated this ADAR1 p110 promoter in HCC cells with/without shAR (Figure 5d). As the three predicted AREs distributed within 200 bp, which prevented the ChIP assays from fine mapping of the corresponding ARE, we mutated these AREs individually (Figure 5e) and performed reporter assays. The results indicated that the ARE1 and ARE2, which may form the canonical ARE-binding site, were responsible for AR-induced activation of the ADAR1 p110 promoter (Figure 5f).

### AR/ADAR1/CircARSP91 axis influences HCC progression

**Figure 6:**
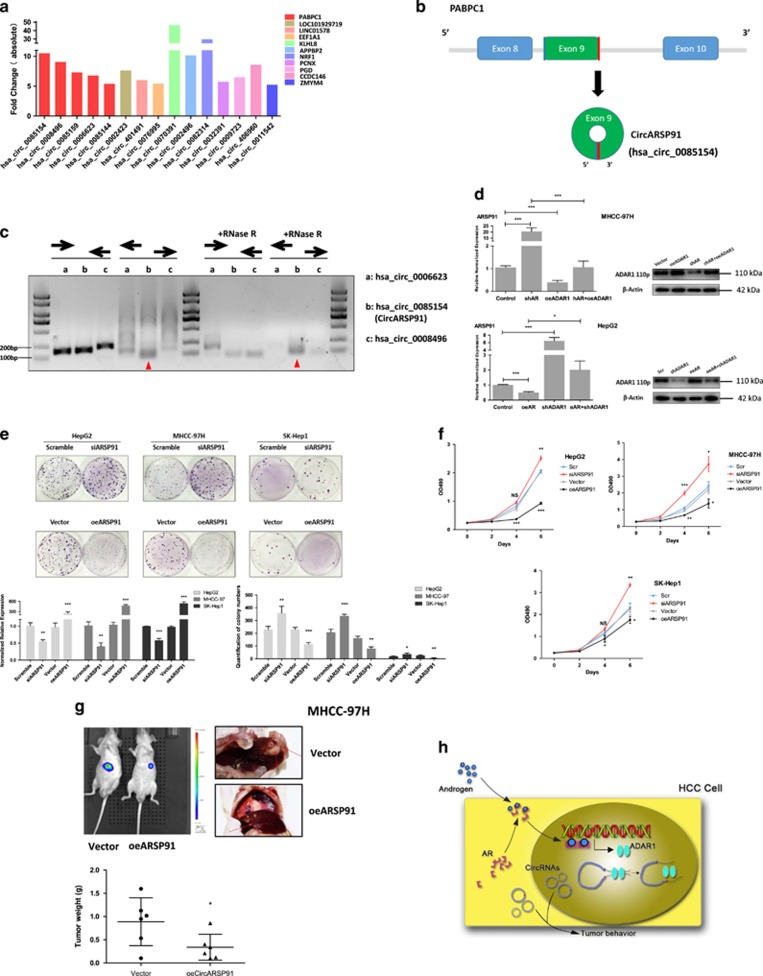
CircARSP91 is regulated by AR/ADAR1 pathway and influences HCC tumorigenesis. (**a**) We listed all the candidates that were upregulated more than fivefold as revealed by circRNA microarray after AR knocking down in MHCC-97h cells. (**b**) Schematic diagram for Circ-ARSP91 formation. (**c**) RNase R was used to treat circRNA candidates to determine the circularity of the RNA. Only CircARSP91 (hsa_circ_0085154) was found to be resistant to enzyme digestion. Inward pointing arrow means linear RNA detection primer whereas outward one means circRNA detection primer. (**d**) RT-qPCR to verify CircARSP91 could be suppressed by the AR/ADAR1 pathway in MHCC-97h cells (upper panel) and in HepG2 cells (lower panel). Right panel showed the knocking down and overexpression efficiency. (**e**) We knocked down or overexpressed CircARSP91 in HCC cell lines (left lower panel showed the knocking down and overexpression efficiency in RT-qPCR) and tested its function on tumor growth with colony-formation assays (upper panel). The quantification was performed and shown in right lower panel. (**f**) We tested the influence of circARSP91 on cell proliferation with MTT assay. The cells were cultured up to 6 days and detected every 2 days after seeding. (**g**) MHCC-97h cells with CircARSP91 overexpressed were orthotopically implanted in nude mice (6 mice in each group) and tumor growth monitored by IVIS. Upper left panel shows representative image of tumor comparison using IVIS, and upper right panel for the orthotopic tumor after killing the mice (red arrow marks primary tumor). Lower panel is the quantitation of tumor weights. (**h**) Schematic diagram of circRNA suppression induced by AR/ADAR1. Data shown are mean±S.D. The siARSP91 was group compared with Scramble (Scr) group, and oeARSP91 group was compared with Vector group. *P<0.05, **P<0.01 and ***P<0.001. ←→, linear RNA detection primer; →←, circRNA detection primer

To examine the biological consequences of AR/ADAR1 expression in HCC development, we focused on 5 circRNAs that originated from PABPC1 (polyadenylate-binding protein 1) gene that were all upregulated more than five-fold after knocking down AR (Figure 6a), whereas AR did not regulate PABPC1 transcript level (Supplementary Figure S4a). One of them, hsa_circ_0085154 that originated from exon 9 appeared to be a true circRNA as it is resistant to RNase R digestion with a clear expression in HCC cell lines (Figures 6b and c and Supplementary Figure S4b). In addition, hsa_circ_0085154 expression could be suppressed by AR in HCC cell lines in an ADAR1-dependent manner (Figure 6d). This particular circRNA is quite small in comparison to ciRS-7 (circular RNA sponge for miR-7, 1485 bp) at 91 bp in length (Supplementary Figure S4c). We renamed it as CircARSP91 (circRNA of AR suppressed PABPC1 91bp). Colony formation and MTT assays revealed that CircARSP91 acted as a tumor suppressor without influence on cell invasion (Figures 6e and f and Supplementary Figure S4d). Consistently, HCC cells with ectopic expression of CircARSP91 displayed a dramatic reduction in tumor growth in an *in vivo* orthotopic injection model of HCC (Figure 6g and Supplementary Figure S4e). These results support a role of AR/ADAR1/CircARSP91 signaling axis in regulating HCC progression (Figure 6h).

## Discussion

In this study we reported a biased expression profile of circRNAs influenced by sex hormone receptors, and such differential profile may trigger a series of consequences and change of HCC biological behaviors. These findings contributed to a complete understanding of the mechanisms underlying the gender disparity in initiation and progression of HCC.^10^ As circRNA distribution and expression showed a complex tissue or cell-type and developmental stage-specific pattern with brain having a significant greater fraction of circRNA junction reads than other organs,^30^ our results provided a unique regulation of circRNA expression in liver cancer through post-transcriptional control of circularization efficiency. Moreover, this regulation seems to only exist in HCC whereas it is much less pronounced in normal liver cells and other tissue type, reflecting potentially a specific role of AR/ADAR1-regulated expression of circRNA in liver cancer progression. Unfortunately, the full biological significance of the change of circRNA expression pattern by the sex hormone receptor is not known, although we indeed identified a novel circular RNA that is important for HCC development both *in vitro* and *in vivo*.

Both *cis*-elements and *trans*-factors could influence the biogenesis of circRNAs. For *cis*-elements, it is believed the sequences upstream and downstream of circRNAs are critical for its formation and among them, Alu repeats and reverse complementary matches (RCMs) have been well documented.^19, 31, 32, 33^ Regarding the *trans*-factors, we successfully linked a formerly reported negative regulator, ADAR1, with AR in HCC and elucidated the source of circRNA expression bias.^19^ Another RNA-binding protein, QKI, seems not to participate in the AR-related biogenesis of circRNAs based on our data.^33^

ADAR1 has been shown to be critical during cancer development via abnormal A-to-I RNA editing^26^ and this function in HCC has also been documented.^18^ Our results imply the AR/ADAR1/circRNA pathway is a novel functional consequence of ADAR1 expression in addition to A-to-I RNA editing, likely critical for HCC development. The ADAR1 transcriptional regulation by AR also provided a new angle to examine AR functions in HCC in addition to those reported.^11, 34^ Our studies strongly support ADAR1 as a tumor promoter in HCC, consistent with the report of Chan *et al.*^18^ In addition, when the follow-up time extends to 120 months, the grouping mode influenced the results. The abnormal elevation of ADAR1 that exceeds adjacent normal tissue (T–N>0) could predict a worse prognosis more effectively than other indications. Based on these evidences, our extended patient follow-up as well as different analytical metrics support the use of ADAR1 staining as a more accurate molecular diagnosis and prognosis marker for HCC.

The canonical ARE structure usually includes two parts of 9 bp sequences spaced by 3 nucleotides.^35^ The ADAR1 promoter computed prediction could only give a general range of possible binding sites, and mutation tests also supported that first and second AREs worked together, and therefore we believed AR binds to a canonical ARE to activate ADAR1 transcription.

The function of circRNAs has been closely linked to the ability to act as a ‘sponge’ of miRNA, thus blocking miRNA regulation of target genes.^12, 35, 36^ We applied a similar analysis on CircARSP91; however, the candidate miRNAs appear to be largely tumor suppressors or nontumor related (Supplementary Figure S4f).^37, 38, 39^ Indeed, it has been argued that sponging circRNAs are exceptional whereas most circRNAs do not have enough miRNA-binding sites to function as sponges according to bioinformatic analysis.^40, 41^ In addition to miRNA sponge, circRNA could also bind to proteins and influence their functions. A newly reported example is circ-Foxo3 that binds to the cyclin-dependent kinase 2 (CDK1) and cyclin-dependent kinase inhibitor 1 (p21)^42^ to regulate cell cycle progression. At this point, the mechanism of how CircARSP91 regulates HCC growth remains to be determined.

## Materials and methods

### Cell culture and transfection

The MHCC-97h, LM3 and LO2 cells were maintained in DMEM (Invitrogen, Grand Island, NY, USA) with 10% fetal bovine serum (FBS), 1% glutamine and 1% penicillin/streptomycin. The SK-Hep1, HepG2, Huh7 and SD cells were maintained in MEM (Invitrogen) with 10% fetal bovine serum (FBS), 1% glutamine and 1% penicillin/streptomycin. The SK-Hep1 and HepG2 were obtained from ATCC (Manassas, VA, USA) and authenticated by China Center for Type Culture Collection (Wuhan, China) in 2015. The MHCC-97h, LM3 and Huh7 cells were kindly provided by Professor Zheng Shusen (Hangzhou, China).^43^ All the experiments were performed within 3 months of resuscitation and the cell passage was <15 generations when initially purchased. All cell lines were cultured in a 5% (v/v) CO_2_ humidified incubator at 37 °C. For androgen free media preparation, dextran-coated charcoal (C6241, Sigma, Shanghai, China) was used to pretreat FBS overnight. Then, the charcoal was filtrated and charcoaled FBS was collected for further experiments.

Cells with stable expression were established based on a previous report.^11^ Briefly, HEK-293T cells were transfected with the core plasmid (pWPI, pLKO1 or pCDH), with the psAX2 packaging plasmid and pMD2G envelope plasmid for 48 h to obtain the lentivirus supernatant that was frozen at −80 °C for later infection to produce stable polyclonal populations.

Our siRNAs targeting CircARSP91 were designed and generated by RiboBio (Guangzhou, China). The knockdown efficiency was determined and we used si-CircARSP91 #1 for further experiments (Supplementary Figure S1a). For the luciferase reporter assays and siRNA interference assays, cells were transfected using LipofectAMINE 3000 (Invitrogen) reverse transfection protocol according to the manufacturer’s instructions. See Supplementary Table S1 for detailed sequence information.

### Quantitative real-time PCR analysis

Total RNAs were isolated using Trizol reagent (Invitrogen), either from cultured cells or frozen tissue of primary tumors and adjacent non-tumor liver tissues. Total RNA (1 *μ*g) was subjected to reverse transcription using Superscript III transcriptase (Invitrogen). RT-qPCR was conducted using a Bio-Rad CFX96 system (Bio-Rad, Hercules, CA, USA) with SYBR green to determine the mRNA expression level of a gene of interest. Expression levels were normalized to the expression of GAPDH mRNA. The detecting primers for circRNAs were obtained from previous reports^21, 22^ or designed based on its head-to-tail junction (see Supplementary Table S1 for detailed sequences).

### Circular RNA microarray

MHCC-97h AR knockdown cells and LO2 AR-overexpressed cells were established and total RNA was extracted according to Circular RNA microarray manufacturer’s standard protocols (KangChen Bio-Tech (Shanghai, China)) including purifying RNA, transcribing into fluorescent cRNA and then hybridizing onto the Human circRNA Arrays (Arraystar v2.0, Rockville, MD, USA). Finally, the hybridized slides were washed, fixed and scanned to images by the GenePix 4000B (Molecular Devices, Sunnyvale, CA, USA), and the data collection was performed and analyzed using corresponding software. The raw data were quantile normalized and further data analysis was performed with R software package, GeneSpring GX (Agilent Technologies, Santa Clara, CA, USA) and gene expression dynamics inspector (GEDI). The statistical significance of differentially regulated circRNAs between the AR knockdown group and control group was identified through *P*-value and FDR filtering. Significant differentially expressed transcripts were retained by screening for fold change ≥2.0, *P*<0.05 and FDR <0.05. Hierarchical clustering was performed to generate an overview of the characteristics of expression profiles based on values of all expressed transcripts and significantly differentially expressed transcripts.

### Western blot analysis

Cells or frozen tissues were lysed in RIPA buffer and proteins (60 *μ*g) were separated on 10–12% SDS/PAGE gel and then transferred onto PVDF membranes (Millipore, Billerica, MA, USA). After blocking, membranes were incubated with appropriate dilutions of specific primary antibodies followed by incubation with HRP-conjugated secondary antibodies and visualization using ECL system (Thermo Fisher Scientific, Rochester, NY, USA). Anti-*β*-ACTIN (1 : 1000, c4) and anti-AR (1 : 1000, N20) antibodies were purchased from Santa Cruz Biotechnology (Santa Cruz, CA, USA). Anti-ADAR1 (1 : 1000, monoclonal, ab126745) antibody, anti-ADAR1 (1 : 1000, polyclonal, ab88574), anti-QKI (1 : 1000, ab126742) were purchased from Abcam (Shanghai, China). Anti-ADAR2 (1 : 500, ENT0119) antibody was purchased from Elabscience Biotechnology (Bethesda, MD, USA).

### Plasmid construction and luciferase assays

The ADAR1 shRNA or scramble sequence was inserted into pLKO1 vector for lentivirus generation. The ADAR1 p110 overexpression plasmid (Z3143) was purchased from GeneCopoeia (Rockville, MD, USA) and converted into pWPI vector by the Gibson assembly method (Supplementary Figure S1b. A promoter of ADAR1 p110 was obtained from genomic DNA of 293T cell by Phusion High-Fidelity DNA Polymerase (NEB, Beverly, NY, USA) and ligated into pGL3-basic vector (Promega, Madison, WI, USA) by the Gibson assembly method. For the ARE mutation, quickchange was used according to the manufacturer’s instruction. For the generation of CircARSP91 (hsa_circ_0085154) overexpression plasmid, we added 300 bp upstream and 300 bp downstream sequence to CircARSP91 nonlinear splice site (total 691 bp). Then, this fragment was inserted into pCDH-CMV-MCS-EF1-GFP+Puro (Geneseed Biotech, Guangzhou, China) to obtain the pCDH-CircARSP91.^12^ Overexpression of CircARSP91 was verified (Supplementary Figure S1C). See Supplementary Table S1 for detailed sequences.

For luciferase assays, cells were plated in 24-well plates with transfection using Lipofectamine3000 (Invitrogen) according to the manufacturer’s instruction. pRL-TK was used as the internal control. Luciferase activity was measured by Dual-Luciferase Assay (Promega) according to the manufacturer’s manual.

### Patient selection and IHC staining

Archived formalin-fixed, paraffin-embedded HCC samples with corresponding adjacent nontumor tissue were obtained from 83 patients undergoing initial hepatectomy from January 2002 to December 2006 in Sir Run Run Shaw Hospital (SRRSH), School of Medicine, Zhejiang University, China (Table 1). All patients were monitored with a 10-year follow-up. The follow-up period was defined as the interval from the date of surgery to the date of recurrence or death. The last follow-up was updated on 20 October 2016. Patients alive at the end of follow-up were censused. Disease-free survival (DFS) was defined as the interval from the date of surgery until the detection of tumor recurrence. Patients without signs of recurrence at the end of follow-up were censused. Patients who died from diseases other than HCC or unexpected events were excluded from the study cohort.

For RT-qPCR and western blot analysis, an additional 14 paired fresh-frozen HCC tissues and corresponding noncancerous tissues were obtained from HCC patients undergoing initial hepatectomy from August 2014 to December 2014 in SRRSH.

The IHC slides of all 83 patients used for ADAR1 scoring were reviewed by two pathologists in a double-blind manner. The staining results were measured semiquantitatively on a scale of (−), (+), (++) and (+++). A stain was scored as follows: (−), there is <50% staining of nuclear ADAR1 in any of the tumor cells/field or <10% staining of nuclear ADAR1 in any of the normal liver cells/field; (+), there is nuclear ADAR1 staining in 50 to 70% of the tumor cells with any intensity, or 10 to 30% staining of the normal liver cells with any intensity; (++), there is staining in >70% of the tumor cells with moderate intensity of nuclear ADAR1, or 30 to 50% staining of the normal liver cells with any intensity; and (+++), there is staining in >70% of the tumor cells nuclei with strong intensity of ADAR1, or >50% staining of the normal liver cells with any intensity. Representative examples of (−), (+), (++) and (+++) IHC staining for AR and ADAR1 are demonstrated in Figure 4a. IHC stains were performed using the standard streptavidin-biotin-peroxidase immunostaining procedure. The antibodies used for anti-ADAR (polyclone, ab88574) were the same as used with western blot with the concentration raised to 1 : 300.

### ChIP assay

Cell lysates were precleared sequentially with normal rabbit IgG (sc-2027, Santa Cruz Biotechnology) and protein A-agarose. Anti-AR antibody from Santa Cruz (2.0 *μ*g) was added to the cell lysates and incubated at 4 °C overnight. IgG was used as the negative control. Specific primer sets designed to amplify a target sequence within the human ADAR1 promoter are listed in the Supplementary Table S1. PCR products were analyzed by agarose gel electrophoresis.

### *In vivo* orthotopic tumor model

A total of 12 male 6–8-week-old nude mice were used. MHCC-97h cells were engineered to express luciferase reporter gene (PCDNA3.0-luciferase) by stable transfection and the positive stable clones were selected with G418 and expanded in culture. After that, stable overexpressing CircARSP91 or control vector MHCC-97h cells were established. Two groups of 12 mice each were injected with HCC cells (MHCC-97h oeARSP91 or MHCC-97h vector, 2 × 10^6^ of luciferase-expressing cells, as a mixture with Matrigel, 1 : 1) into the left lobe of liver. Tumor formation and metastasis were monitored by using a Fluorescent Imager (IVIS Spectrum, Caliper Life Sciences, Hopkinton, MA, USA) starting 2 weeks after tumor injection and using mouse tail vein injection of 150 mg/kg Luciferin. Mice injected with HCC cells were killed after 8 weeks and liver tumors were isolated for further testing.

All animal experiments were performed humanely in compliance with guidelines reviewed by the animal ethics committee of the Biological Resource Centre of the Agency for Science, Technology and Research, Zhejiang University.

### MTT assay and colony formation assay

Stable transfected cells or transient transfected cells (4 × 10^3^) were seeded on a 96-well plate with 3 replicate wells and allowed to incubate up to 6 days. After incubation, cell viability was assessed at 2, 4 and 6 days utilizing the tetrazolium-based MTT colorimetric assay (CellTiter 96-well cell proliferation assay kit; Promega) according to the manufacturer’s instructions. All experiments were performed at least in triplicate on three separate occasions.

In colony-formation assays, cells were plated in 10 cm plates at a density of 1 × 10^3^ cells/plate. At least 3 plates were used as replicates. Cells were maintained in proper culture medium with 10% FBS. All cell lines were cultured in a 5% (v/v) CO_2_ humidified incubator at 37 °C for 20 days. After that, the plates were stained by 0.1% crystal violet.

### Invasion assay

The invasion capability of transfected HCC cancer cells was determined by the transwell (BD Falcon, Franklin Lakes, NJ, USA) assay. Before seeding the cells, 10 ml of Matrigel (BD, Inc.) was dissolved in 50 ml serum-free proper culture medium, applied to upper chamber of 8 mm pore size polycarbonate membrane filters (Corning, Inc., Corning, NY, USA) and put into the incubator for 2 h. Transfected cancer cells were seeded with serum-free medium into the upper chamber at 1 × 10^5^ cells/well, and the bottom chamber of the apparatus contained culture medium with 10% FBS, and then incubated for 48 h at 37 °C. Following incubation, the invaded cells that attached to the lower surface of the membrane were fixed by 4% paraformaldehyde and stained with 0.1% crystal violet.

### Statistical analysis

Data are expressed as mean±S.D. from at least three independent experiments. Statistical analyses involved paired and unpaired *t*-test, one-way ANOVA and Spearman’s correlation with SPSS 17.0 (SPSS Inc., Chicago, IL, USA).

Kaplan–Meier method with log-rank test was used to compare patients’ survival between subgroups. The patients were divided into subgroups as follows: Negative, ADAR1 staining shows (−) in tumor tissues; Positive, ADAR1 staining shows (+), (++) or (+++) in tumor tissues; T-N≤0, tumor ADAR1 staining scores equally or less than normal tissues; T-N>0, tumor ADAR1 staining scores higher than normal tissues. *P*<0.05 was considered statistically significant.

## Conclusions

In summary, our findings indicate a novel feature of circRNA expression pattern that may be suppressed by the AR/ADAR1 pathway in HCC. This biased distribution of circRNA could help to explain the obvious gender imbalance of HCC from a new perspective. Our analysis led to the identification of a novel circular RNA, CircARSP91, as a potent repressor of HCC tumor formation both *in vitro* and *in vivo*, establishing a foundation to search novel therapies of HCC through reactivation of this circular RNA.

## Figures and Tables

**Table 1:** Clinicopathological analyses of the differentially expressed ADAR1 of 83 primary hepatocellular carcinoma patients

**Variables**		**ADAR**		***P*****-value**
		**Negative**	**Positive**	
Age, years^a^	≤60	12 (75.0)	55 (82.1)	0.498
	>60	4 (25.0)	12 (17.9)	
Gender^a^	Female	2 (12.5)	16 (23.9)	0.503
	Male	14 (87.5)	51 (76.1)	
HBV^a^	No	3 (18.8)	11 (16.4)	1.000
	Yes	13 (81.3)	56 (83.6)	
Cirrhosis	No	7 (43.8)	33 (49.3)	0.692
	Yes	9 (56.3)	34 (50.7)	
AFP, ng/ml	≤400	10 (62.5)	42 (62.7)	0.989
	>400	6 (37.5)	25 (37.3)	
Tumor size, cm	≤5	8 (50.0)	43 (64.2)	0.295
	>5	8 (50.0)	24 (35.8)	
Recurrence	No	10 (62.5)	27 (40.3)	0.108
	Yes	6 (37.5)	40 (59.7)	

Abbreviations: ADAR1, adenosine deaminases that act on RNA; AFP, *α-*fetoprotein; HbsAg, hepatitis B surface antigen

Negative means ADAR1 IHC scored as –,whereas positive scored as +, ++ or +++

a Fisher’s exact test was used
