# Supplementary material for: Circular RNA expression is suppressed by androgen receptor (AR)-regulated adenosine deaminase that acts on RNA (ADAR1) in human hepatocellular carcinoma
Source: Cell Death Dis. 2017 Nov 16;8(11):e3171–. doi: 10.1038/cddis.2017.556 (PMC5775411; doi:10.1038/cddis.2017.556)
Supplement: Supplementary Figures [file cddis2017556x1.ppt]

## Slide 1
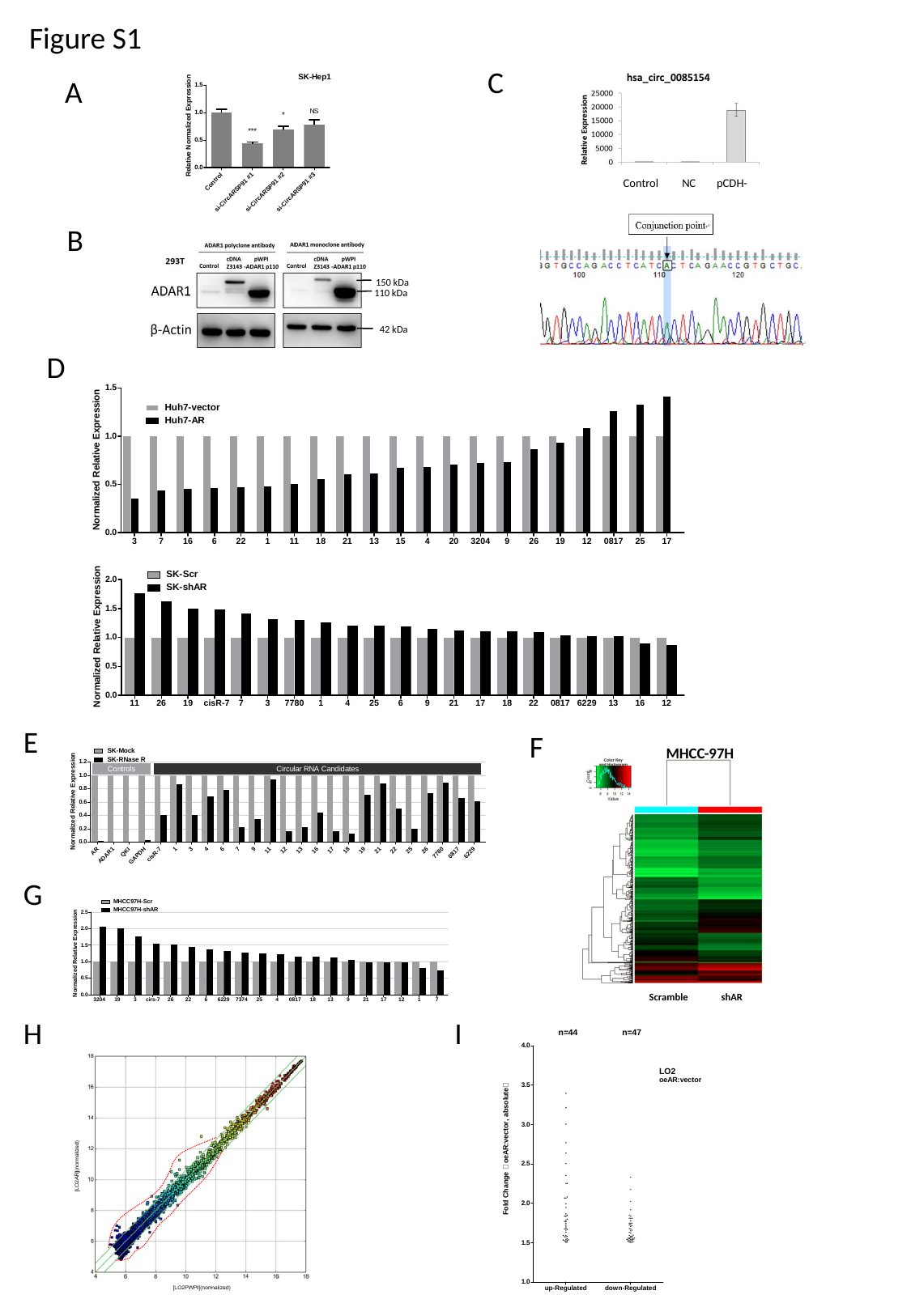

Figure S1
C
Control NC pCDH-
 0085154
A
B
150 kDa
110 kDa
 42 kDa
D
E
F
MHCC-97H
Scramble
shAR
G
H
I

## Slide 2
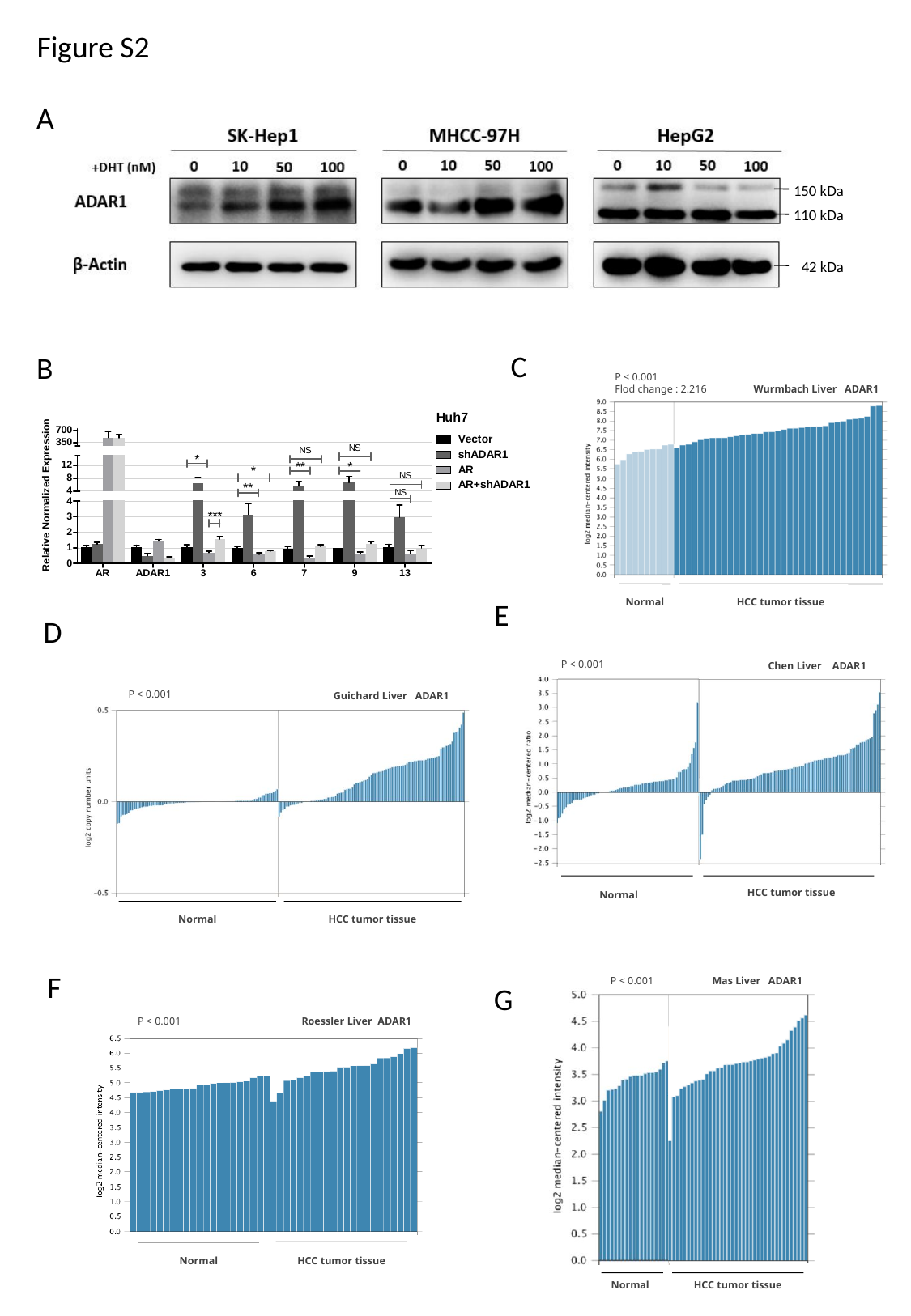

Figure S2
A
150 kDa
110 kDa
 42 kDa
C
B
P < 0.001
Flod change : 2.216
Wurmbach Liver ADAR1
Normal
HCC tumor tissue
E
D
P < 0.001
Chen Liver ADAR1
HCC tumor tissue
Normal
P < 0.001
Guichard Liver ADAR1
Normal
HCC tumor tissue
F
P < 0.001
Mas Liver ADAR1
Normal
HCC tumor tissue
G
P < 0.001
 Roessler Liver ADAR1
Normal
HCC tumor tissue

## Slide 3
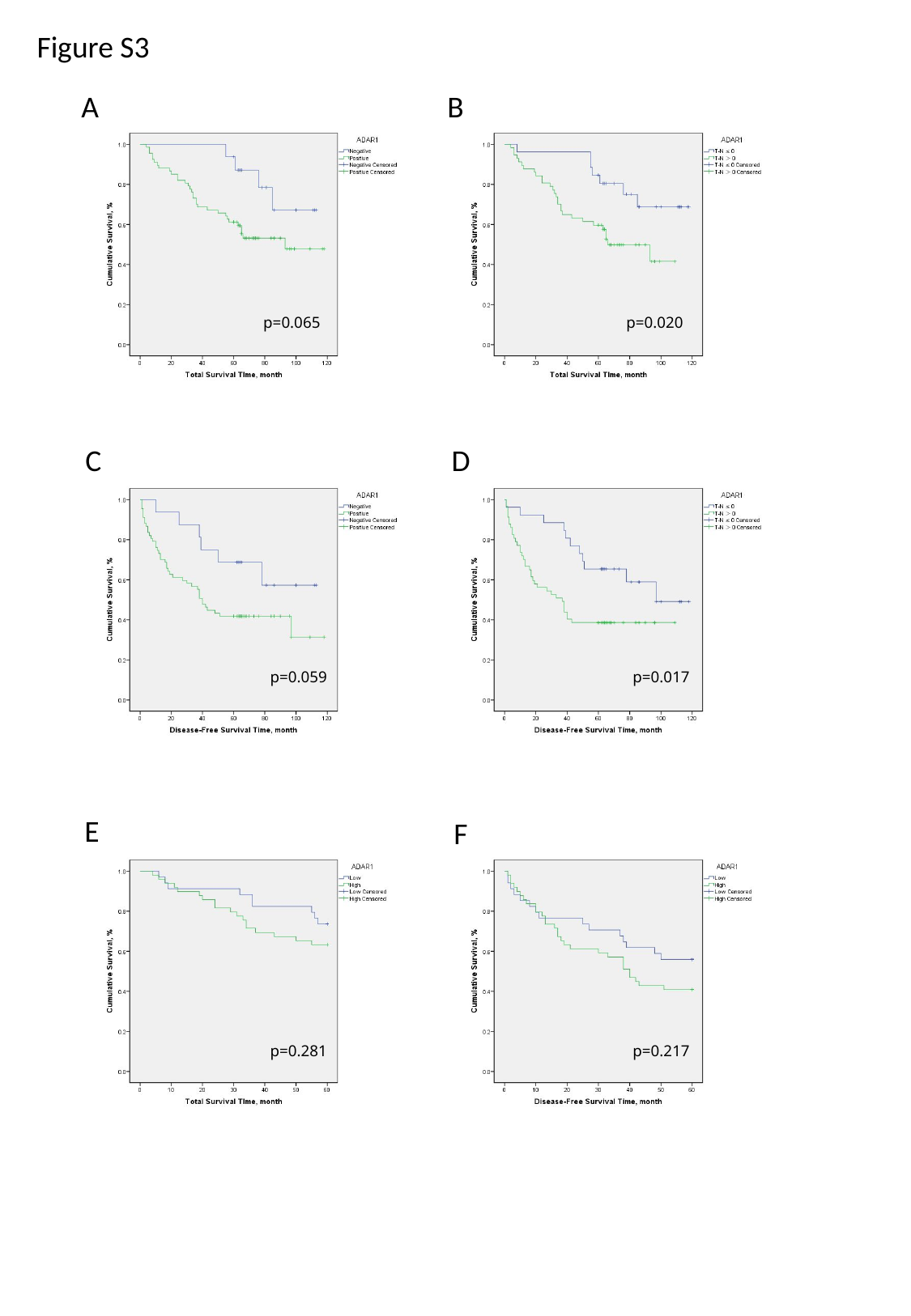

Figure S3
A
B
p=0.065
p=0.020
C
D
p=0.059
p=0.017
E
F
p=0.281
p=0.217

## Slide 4
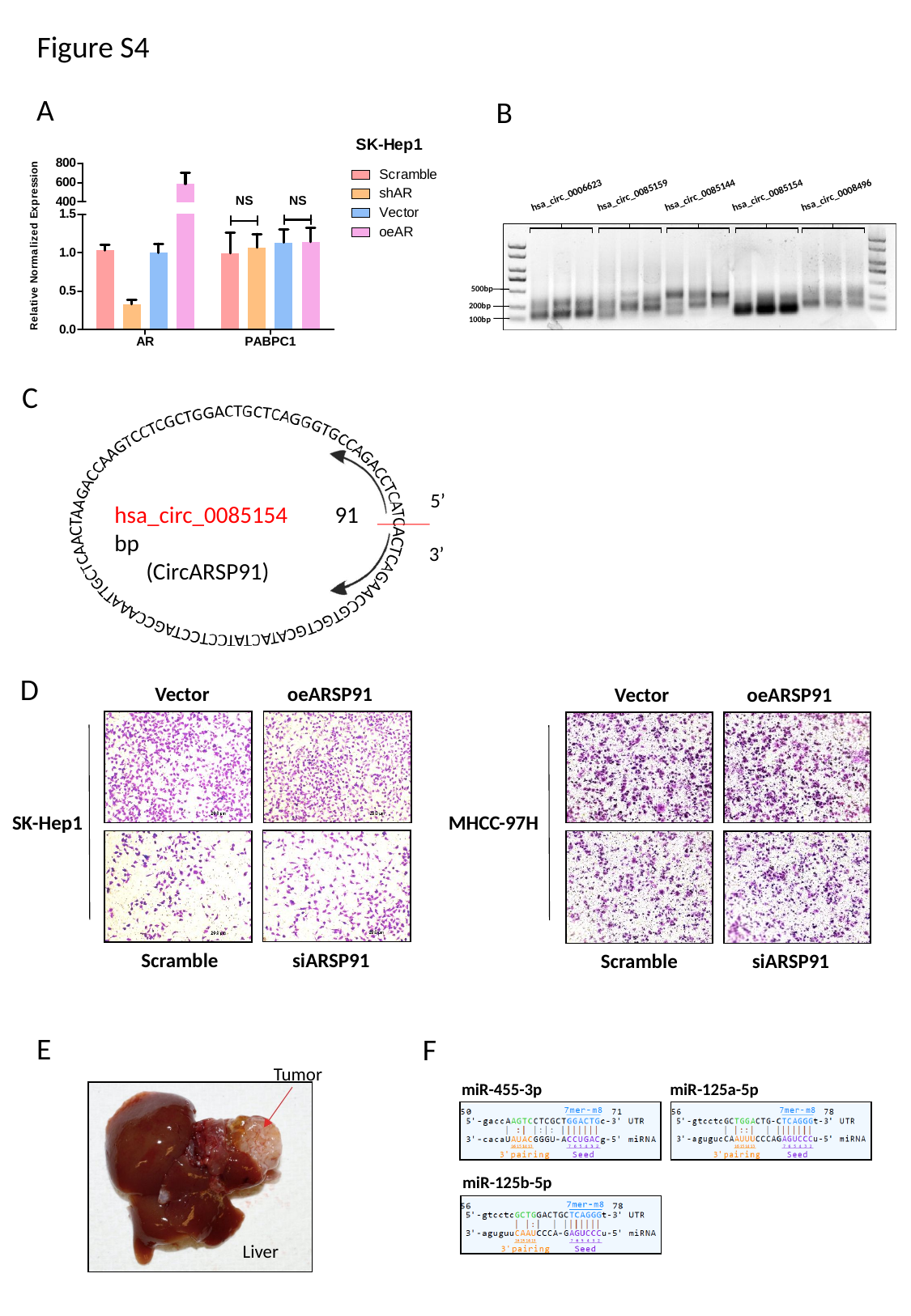

Figure S4
A
B
hsa_circ_0006623
hsa_circ_0008496
hsa_circ_0085159
hsa_circ_0085144
hsa_circ_0085154
500bp
200bp
100bp
C
hsa_circ_0085154：91 bp
 (CircARSP91)
5’
3’
D
Vector
oeARSP91
SK-Hep1
Scramble
siARSP91
Vector
oeARSP91
MHCC-97H
Scramble
siARSP91
E
F
Tumor
Liver
miR-455-3p
miR-125a-5p
miR-125b-5p

## Slide 5
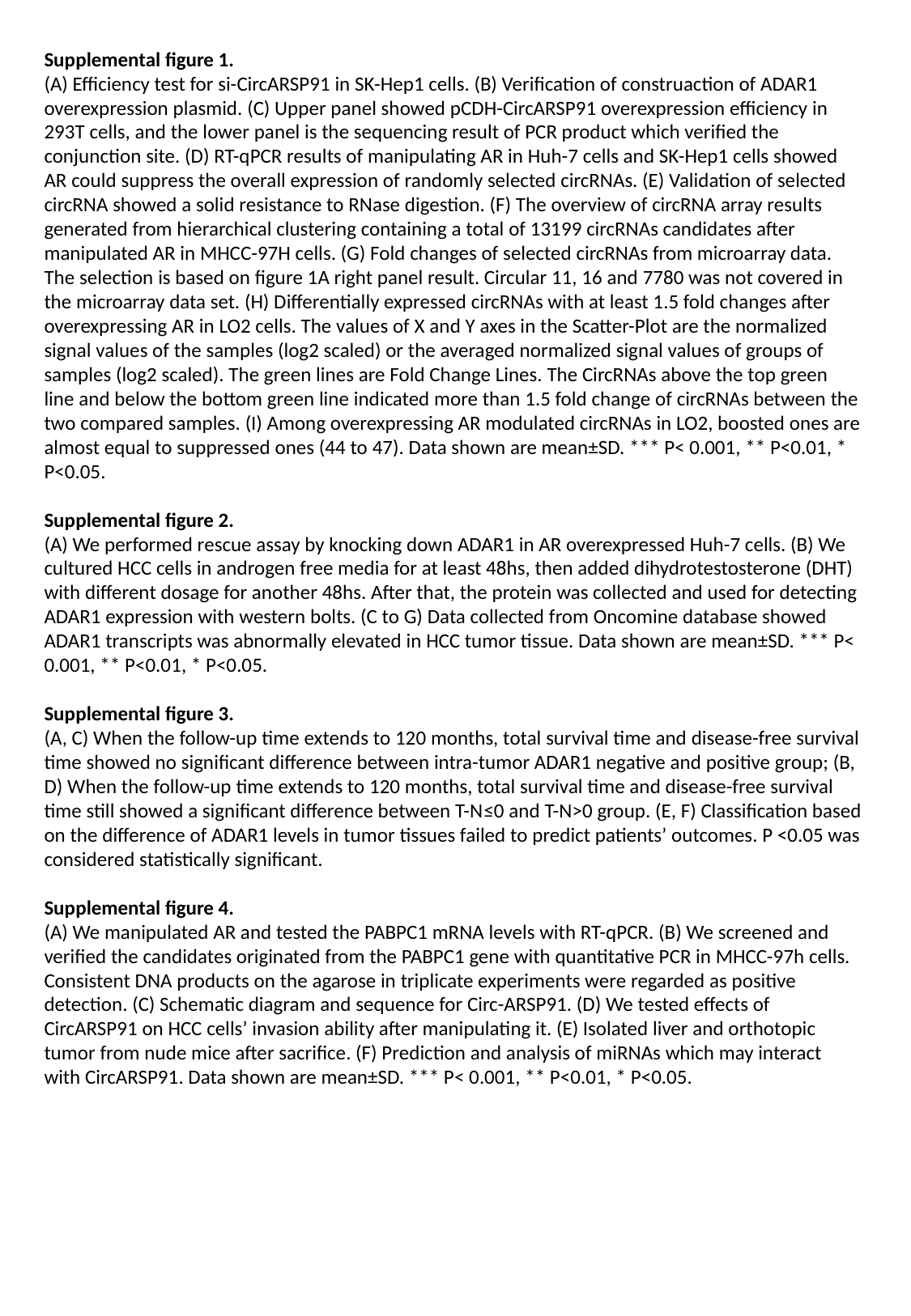

Supplemental figure 1.
(A) Efficiency test for si-CircARSP91 in SK-Hep1 cells. (B) Verification of construaction of ADAR1 overexpression plasmid. (C) Upper panel showed pCDH-CircARSP91 overexpression efficiency in 293T cells, and the lower panel is the sequencing result of PCR product which verified the conjunction site. (D) RT-qPCR results of manipulating AR in Huh-7 cells and SK-Hep1 cells showed AR could suppress the overall expression of randomly selected circRNAs. (E) Validation of selected circRNA showed a solid resistance to RNase digestion. (F) The overview of circRNA array results generated from hierarchical clustering containing a total of 13199 circRNAs candidates after manipulated AR in MHCC-97H cells. (G) Fold changes of selected circRNAs from microarray data. The selection is based on figure 1A right panel result. Circular 11, 16 and 7780 was not covered in the microarray data set. (H) Differentially expressed circRNAs with at least 1.5 fold changes after overexpressing AR in LO2 cells. The values of X and Y axes in the Scatter-Plot are the normalized signal values of the samples (log2 scaled) or the averaged normalized signal values of groups of samples (log2 scaled). The green lines are Fold Change Lines. The CircRNAs above the top green line and below the bottom green line indicated more than 1.5 fold change of circRNAs between the two compared samples. (I) Among overexpressing AR modulated circRNAs in LO2, boosted ones are almost equal to suppressed ones (44 to 47). Data shown are mean±SD. *** P< 0.001, ** P<0.01, * P<0.05.
Supplemental figure 2.
(A) We performed rescue assay by knocking down ADAR1 in AR overexpressed Huh-7 cells. (B) We cultured HCC cells in androgen free media for at least 48hs, then added dihydrotestosterone (DHT) with different dosage for another 48hs. After that, the protein was collected and used for detecting ADAR1 expression with western bolts. (C to G) Data collected from Oncomine database showed ADAR1 transcripts was abnormally elevated in HCC tumor tissue. Data shown are mean±SD. *** P< 0.001, ** P<0.01, * P<0.05.
Supplemental figure 3.
(A, C) When the follow-up time extends to 120 months, total survival time and disease-free survival time showed no significant difference between intra-tumor ADAR1 negative and positive group; (B, D) When the follow-up time extends to 120 months, total survival time and disease-free survival time still showed a significant difference between T-N≤0 and T-N>0 group. (E, F) Classification based on the difference of ADAR1 levels in tumor tissues failed to predict patients’ outcomes. P <0.05 was considered statistically significant.
Supplemental figure 4.
(A) We manipulated AR and tested the PABPC1 mRNA levels with RT-qPCR. (B) We screened and verified the candidates originated from the PABPC1 gene with quantitative PCR in MHCC-97h cells. Consistent DNA products on the agarose in triplicate experiments were regarded as positive detection. (C) Schematic diagram and sequence for Circ-ARSP91. (D) We tested effects of CircARSP91 on HCC cells’ invasion ability after manipulating it. (E) Isolated liver and orthotopic tumor from nude mice after sacrifice. (F) Prediction and analysis of miRNAs which may interact with CircARSP91. Data shown are mean±SD. *** P< 0.001, ** P<0.01, * P<0.05.
